# Hepatic Vascular Involvement in Adenosine Deaminase 2 Deficiency (DADA2): Case Reports and Literature Review †

**DOI:** 10.3390/diagnostics16020189

**Published:** 2026-01-07

**Authors:** Mihaela Sparchez, Laura Damian, Mihai Adrian Socaciu, Otilia Fufezan, Zeno Sparchez

**Affiliations:** 1 Second Pediatric Discipline, “Iuliu Hatieganu” University of Medicine and Pharmacy, 400177 Cluj-Napoca, Romania; 2 Emergency Clinical Hospital for Children Cluj-Napoca, 4000370 Cluj-Napoca, Romania; 3 Department of Rheumatology, Emergency County Clinical Hospital Cluj, 400006 Cluj-Napoca, Romania; 4 Department of Medical Imaging, “Iuliu Hatieganu” University of Medicine and Pharmacy, 400162 Cluj-Napoca, Romania; 5 Department of Internal Medicine, “Iuliu Hatieganu” University of Medicine and Pharmacy, 400162 Cluj-Napoca, Romania

**Keywords:** deficiency of adenosine deaminase 2, hepatic vascular involvement, nodular regenerative hyperplasia, FNH-like lesions, portal hypertension, vasculitis, immunodeficiency

## Abstract

**Background and Clinical Significance**: Deficiency of Adenosine Deaminase 2 (DADA2) is a rare monogenic vasculopathy characterised by systemic inflammatory and immunodeficiency features. Although neurological and haematological manifestations are well-documented, hepatic vascular involvement remains underappreciated. This report aims to describe the clinical and imaging characteristics of hepatic vascular involvement in a patient with DADA2 and to illustrate the evolution of hepatic lesions during long-term Etanercept therapy. In addition, we provide a synthesis of the available evidence on hepatic manifestations in DADA2, emphasising vascular pathology, clinical presentation, and therapeutic implications. **Case Presentation:** We describe a girl with early-onset DADA2 presenting with recurrent systemic inflammation, hypogammaglobulinaemia, vasculopathy, and two childhood strokes, followed by the development of multiple FNH-like hepatic nodules on CEUS and MRI with persistently elevated GGT. Genetic testing confirmed biallelic ADA2 mutations, and treatment with Etanercept led to sustained clinical stabilisation and marked regression of liver lesions over a nine-year follow-up period. Her older sister, carrying the same mutations, showed a milder phenotype without hepatic involvement but experienced a mesenteric vascular event. **Conclusions**: Large regenerative nodules with an FNH-like appearance on CEUS or MRI have not been previously reported in this setting. In our patient, Etanercept therapy produced a favourable hepatic response, reflected by a significant reduction in both the number and size of the lesions. Our case contributes to the understanding of liver disease in DADA2 and the influence of imaging and treatment on the hepatic manifestations of the condition.

## 1. Introduction

Deficiency of Adenosine Deaminase 2 (DADA2) is a monogenic autoinflammatory and immunodeficiency disorder first described in 2014 [1]. Caused by biallelic mutations in the ADA2 gene (*CECR1*), DADA2 is characterised by systemic vasculopathy, recurrent fever, immunodeficiency, cytopenias, and variable organ involvement [1]. While neurological, haematological, and rheumatological manifestations have been extensively reported, hepatic involvement remains less well-characterised [2,3]. Nevertheless, emerging evidence suggests that hepatic vascular manifestations—including nodular regenerative hyperplasia, hepatoportal sclerosis, portal hypertension, and Budd–Chiari syndrome [4,5]—are an underrecognized but significant component of the DADA2 phenotype.

Meyts and Aksentijevich (2018) [2] and Lee et al. (2022) [3] provided comprehensive reviews of the clinical spectrum of DADA2, highlighting multi-organ vasculopathy but only briefly mentioning hepatic manifestations. More systematic phenotypic surveys, such as those by Barron et al. (2021) [4] and Maccora et al. (2023) [5], have explicitly documented hepatic involvement, reporting hepatomegaly, elevated liver enzymes, and biopsy findings of vascular pathology.

The vasculopathy associated with this rare monogenic disorder primarily affects small- and medium-sized arteries. Biallelic loss-of-function mutations in the ADA2 gene cause immune dysregulation, leading to a skewed macrophage polarisation towards a pro-inflammatory M1 phenotype, endothelial dysfunction, and increased TNF-α production. This results in inflammation of the vessel walls, necrosis, and subsequent vascular occlusion or aneurysm formation [2,3].

Histological data from cohorts and case series reveal a recurring pattern of vascular injury in the liver (compromised endothelial integrity, endothelial cellular activation and inflammation) [1]. Barron et al. (2021) [4] reported that 12% of patients developed portal hypertension, with biopsy findings including nodular regenerative hyperplasia (NRH), hepatoportal sclerosis, and portal vasculopathy. Springer et al. (2018) [6] similarly identified NRH as a key lesion, implicating small-vessel injury as a central pathogenic mechanism. Zhou et al. (2014) [1] included patients with liver biopsy evidence of vascular inflammation in their seminal description of DADA2, providing early evidence of hepatic involvement.

Nodular regenerative hyperplasia (NRH) denotes a diffuse transformation of the hepatic parenchyma into multiple small regenerative nodules, characterised by the absence of fibrosis. It involves the entire liver and can lead to non-cirrhotic portal hypertension. This condition probably stems from intrahepatic microvascular injury and hypoperfusion, which cause hepatocyte injury and the formation of regenerative nodules. Histologically, it is characterised by numerous small regenerative nodules separated by compressed hepatic plates, with no fibrous septa [7,8].

In NRH, regenerative nodules typically measure 1–3 mm in diameter, although larger lesions—up to 1–2 cm—may also occur. These have been referred to as large regenerative nodules or multiacinar regenerative nodules, defined histologically as regenerative nodules containing more than one portal tract [9,10]. Some of these larger nodules exhibit imaging characteristics similar to focal nodular hyperplasia (FNH) and are therefore described as FNH-like lesions [11]. According to current literature, there is no clear consensus on whether these lesions represent a variant of large regenerative nodules (LRN) or a distinct pathological entity [11]. FNH-like lesions have been described in children in association with a variety of liver conditions that alter hepatic perfusion and increase arterial inflow, including Budd–Chiari syndrome [11], congestive hepatopathy, Fontan-associated liver disease [12], sinusoidal obstruction syndrome after chemotherapy or hematopoietic stem cell transplantation [13], autoimmune hepatitis [11,14], cavernous transformation of the portal vein, hereditary hemorrhagic telangiectasia, and common variable immunodeficiency [11].

To date, no cases of focal nodular hyperplasia-like (FNH-like) hepatic lesions have been explicitly reported in patients with DADA2.

Mechanistically, hepatic manifestations seem to reflect the systemic vasculopathy linked to DADA2. Endothelial dysfunction, TNF-driven vascular inflammation, and defective perivascular repair mechanisms have been described (Lee et al. 2022) [3]. The liver, with its dual blood supply and dense sinusoidal network, may be particularly vulnerable to microvascular injury, leading to reactive or regenerative lesions and non-cirrhotic portal hypertension.

Literature evidence is extremely sparse on Budd–Chiari syndrome (BCS) associated with DADA2, suggesting it is a rare or underreported condition.

Clinically, hepatic involvement in DADA2 ranges from asymptomatic enzyme abnormalities to overt complications of non-cirrhotic portal hypertension and end-stage liver disease. However, elevated liver enzymes and hepatosplenomegaly are the most common clinical signs of liver-related disease [1,15,16]. Barron et al. (2021) [4] observed that liver fibrosis was detectable by transient elastography in approximately 25% of cases, while 7 of 56 patients developed portal hypertension with clinical consequences such as splenomegaly and varices. Colangelo et al. (2023) [17] described a striking case of Budd–Chiari syndrome, demonstrating that hepatic venous outflow obstruction can also occur. Case series from Iran (Ashari et al. 2023) [18] and other international cohorts have reported hepatomegaly, elevated transaminases, and hepatosplenomegaly in subsets of patients.

The diagnosis of NRH is often difficult because its histopathologic features may be interpreted variably, and most patients lack specific symptoms or laboratory abnormalities [14]. It may, however, be accompanied by slightly elevated ALP and gamma-glutamyltransferase levels, with a gradual increase over the years leading to chronic cholestasis, non-cirrhotic portal hypertension, or cirrhosis [11].

This article aims to synthesise current evidence on hepatic vascular involvement in DADA2, delineate key patterns, and discuss clinical implications, while adding a novel observation to the hepatic phenotype of the disease: large regenerative nodules of focal nodular hyperplasia appearance (FNH-like) in one patient and a favourable evolution of these lesions under anti-TNF therapy.

## 2. Case Presentation

### 2.1. Case 1

The first case involved a girl born to non-consanguineous parents and with no significant pathology in the family.

At 1.66 years of age, she suddenly presented with a high fever, a nonspecific maculopapular rash, arthralgia, and myalgia, accompanied by elevated levels of acute phase reactants and a rapid response to steroids. One year later, these symptoms recurred. A skin biopsy revealed interstitial granulomatous dermatitis associated with vasculopathy.

At the age of 3, she began experiencing recurrent cranial nerve palsies and livedo reticularis, followed by two stroke events at age 4—one ischemic and the other hemorrhagic. Magnetic resonance angiography revealed no signs of cerebral vasculitis, and a brain biopsy performed during the surgical procedure for the hemorrhagic stroke showed only extravasation of erythrocytes and fibrin deposition around small vessels without significant inflammation. The antibody profile was negative, including antinuclear antibodies, antineutrophil cytoplasmic antibodies, autoantibodies associated with autoimmune hepatitis, and antiphospholipid antibodies. Additionally, clotting tests were within the normal range.

Hypogammaglobulinemia with global immunoglobulin deficiency was present from the onset of the disease and persisted over time. She could not be classified as having common variable immunodeficiency (CVID) because her total serum IgG levels were not consistently below 500 mg/dL. As part of a research study, she was also found to have a complete C4B deficiency.

At the age of 5, she developed moderate thrombocytopenia that persisted for the following 3 years.

**Figure 1:**
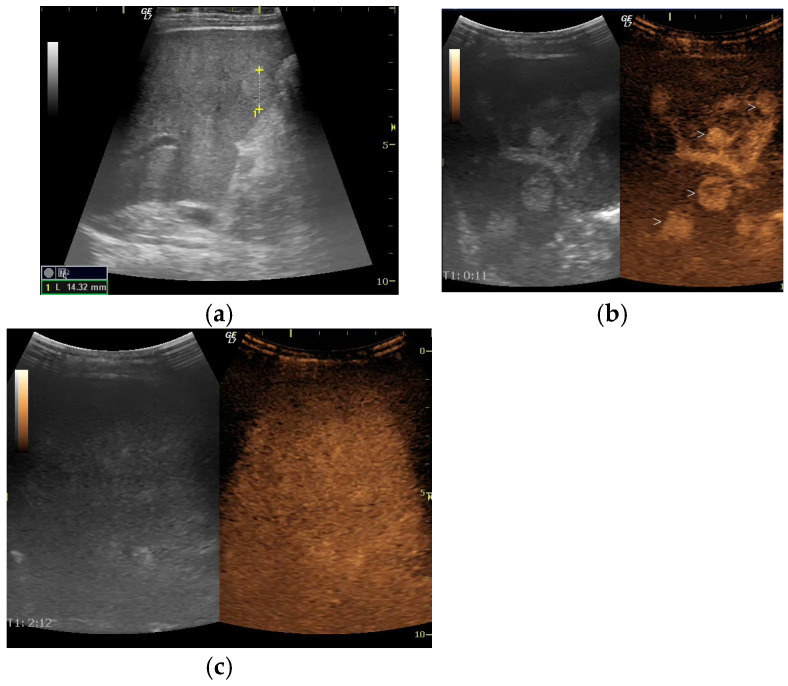
Initial ultrasound (US) of the liver before treatment. (**a**) Grey-scale US showing a hyperechoic nodule in the right lobe; (**b**) Contrast-enhanced ultrasound (CEUS). Arterial phase. Multiple round hyperenhancing nodules in the right lobe (marked with the symbol ‘>’ on the image); (**c**) CEUS in parenchymal phase. The nodules are isoenhanced and no longer detectable.

At 5.5 years of age, ultrasound revealed multiple diffuse hyperechoic hepatic lesions measuring 1–2 cm and mild splenomegaly. On contrast-enhanced ultrasound (CEUS), the lesions exhibited arterial-phase hyperenhancement with centrifugal filling and became isoenhancing during the portal and late phases (Figure 1). These features were interpreted as consistent with large regenerative nodules of focal nodular hyperplasia-like appearance (FNH-like). At this stage, serum GGT levels were elevated and remained persistently high, peaking at 120 U/L. Serum transaminases (AST and ALT) were only slightly elevated, with ALT sometimes remaining within the normal range.

**Figure 2:**
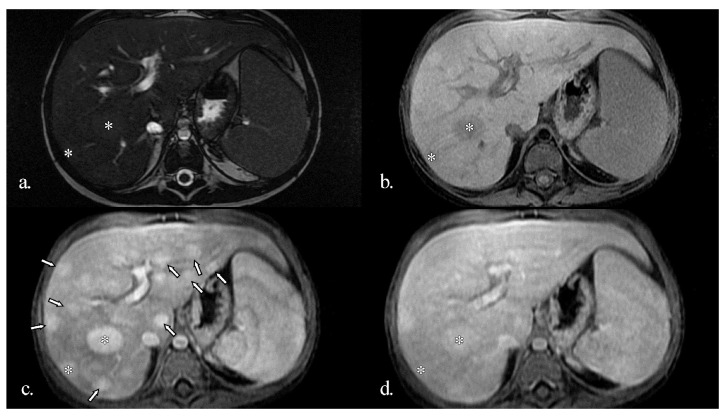
Initial MRI examination of the liver. (**a**) Native T2-weighted; (**b**) Native fat-saturated T1-weighted; Gd-enhanced fat-saturated T1-weighted in the arterial (**c**) and late (**d**) phases. Multiple hyperenhancing focal lesions are visible in the arterial phase (arrows, asterisks), most are isointense on T2w and native T1w images, iso- or hyperintense in the late phase, with no washout. The lesions are relatively similar in size (10 to 25 mm) and imaging features. Two of the lesions (asterisks) are slightly hyperintense on T2w and hypointense on native T1w, while still enhancing after Gd injection.

MRI showed multiple hyperenhancing focal lesions visible in the arterial phase, disseminated throughout the entire hepatic parenchyma; most were isointense on T2w and native T1w images, iso- or hyperintense in the late phase, with no washout. The lesions were relatively similar in size (10–25 mm) and imaging features (Figure 2).

A liver biopsy was not performed at that time due to the increased risk of bleeding given our patient’s thrombocytopenia, the absence of severe liver damage or other risk factors for primary liver malignancies and typical radiologic features of FNH-like nodules.

During the first two years of her condition, only high doses of corticosteroids were effective in controlling the disease manifestations. Treatments such as methotrexate, azathioprine, cyclosporine, and cyclophosphamide proved ineffective. In contrast, mycophenolate mofetil (MMF) combined with high monthly doses of intravenous immunoglobulin led to prompt and sustained improvements in clinical symptoms and acute-phase reactants, allowing for a gradual reduction in steroid dosage. With MMF treatment, no recurrences were observed; however, the FNH-like lesions showed slow progression.

At the age of 8, genetic testing revealed two homozygous pathogenic mutations in the ADA2 gene (*p.G326R* and *p.V458D*). Plasma ADA2 enzyme activity was notably low, measured at 3 mU/mL/h. The patient was commenced on Etanercept, which effectively controlled systemic inflammation, thrombocytopenia, and vascular symptoms. Over the following 9 years, no neurological events occurred. She is hemiparetic due to the previous stroke, and some disease features persist, including hypogammaglobulinemia and livedo reticularis.

**Figure 3:**
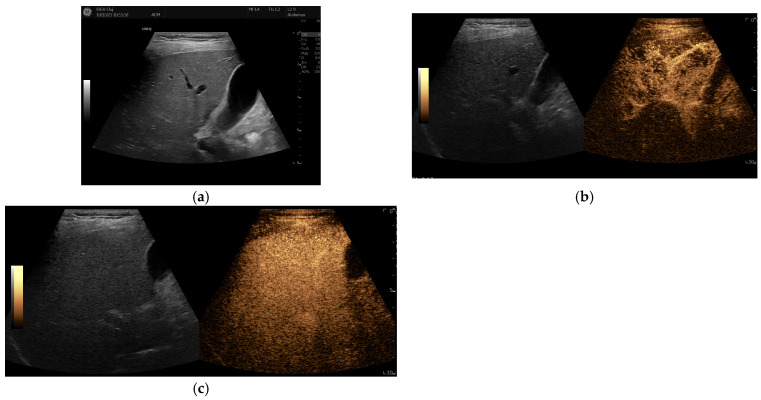
Ultrasound (US) evaluation after 9 years of Etanercept treatment. (**a**) B Mode US shows no evident liver nodules; (**b**) Contrast-enhanced ultrasound (CEUS) arterial phase shows an inhomogeneous pseudonodular enhancement of the right lobe; (**c**) CEUS in parenchymal phase—no hypoenhanced nodules are seen.

**Figure 4:**
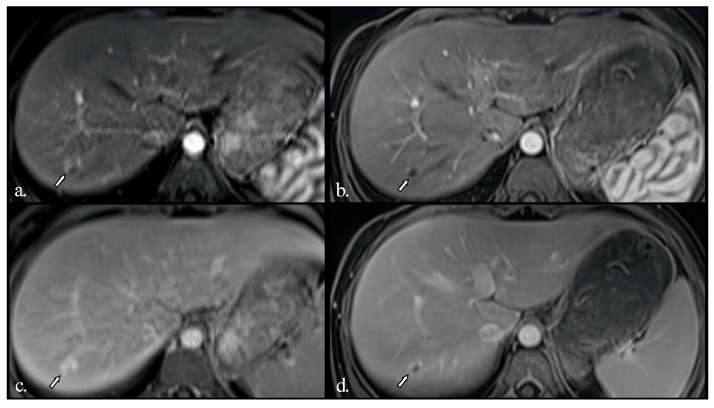
Subsequent MRI examinations after 5 years (**a**,**c**) and 9 years (**b**,**d**) of Etanercept treatment since the initial assessment. Gd-enhanced fat-saturated T1-weighted images in the arterial (**a**,**b**) and late (**c**,**d**) phases. Only one lesion (arrow) remains visible, showing hyperenhancement in the periphery and a central scar with progressive, incomplete filling in the late phases. The lesion has shrunk in the last examination.

Remarkably, under anti-TNF treatment, there has been a significant improvement in liver lesions, as evidenced by reductions in both their size and number on repeated liver imaging (Figure 3 and Figure 4), along with normalisation of GGT levels in subsequent years. Improvement was evident after just one year of treatment and continued to progress until the patient reached 17 years of age. Splenomegaly persisted without evidence of portal hypertension, and liver stiffness on elastography decreased slightly over time (6.1 kPa before Etanercept and 5.6 kPa after treatment).

**Figure 5:**
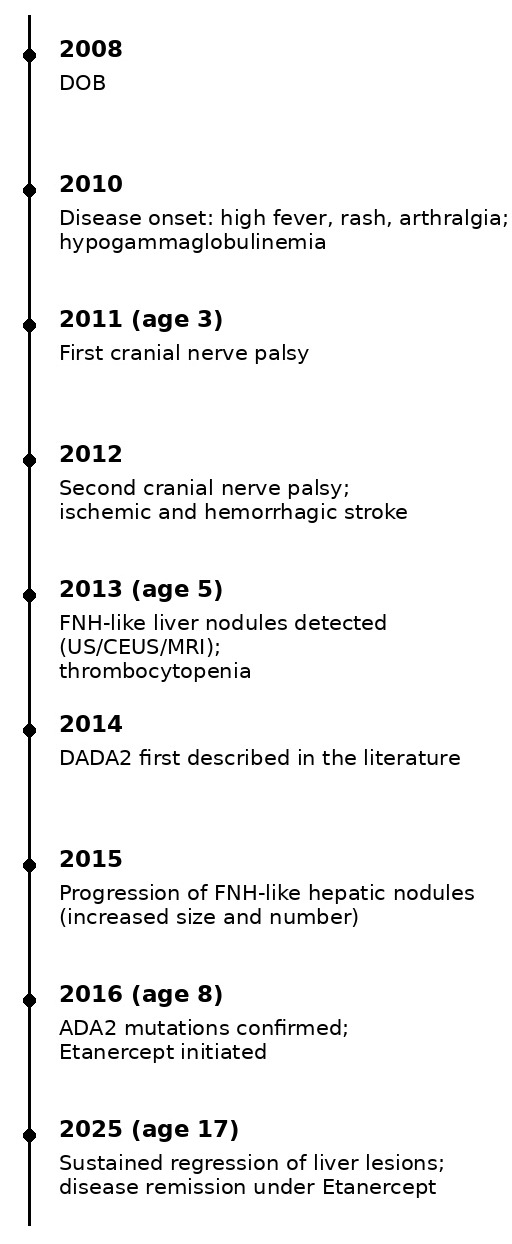
Concise timeline of disease progression, liver involvement, and treatment response in Patient 1 with DADA2.

The timeline (Figure 5) shows the progression of disease manifestations, liver involvement, and treatment response in patient 1.

### 2.2. Case 2

The older sister of the patient described above, who shares the same homozygous pathogenic mutations in the ADA2 gene, exhibited a later onset of clinical symptoms (at 6.5 years old), including persistent fever, myalgia, livedo reticularis, vascular purpura, painful subcutaneous nodules, systemic arterial hypertension, recurrent abdominal pain, and a highly persistent inflammatory response. In her case, the immunodeficiency phenotype appeared milder, with slightly reduced IgM levels and normal IgG and IgA levels. She did not show any hepatic vascular involvement of the disease, nor did she develop neurological symptoms. However, at 7.5 years of age, she experienced a massive intestinal bleed, preceded by acute abdominal pain, considered a vascular complication affecting the mesenteric vessels. The source of the bleeding was not identified on endoscopic and imaging studies. At the last assessment, during ongoing Etanercept treatment, patient 2 was in persistent clinical remission with no new complications.

## 3. Discussion

The literature indicates that hepatic vascular involvement is a significant yet often overlooked aspect of DADA2. While neurological and haematological symptoms are predominant, up to one-third of patients may exhibit biochemical, radiological, or histopathological signs of liver disease. The most consistent observation across reports is nodular regenerative hyperplasia, indicating noncirrhotic portal hypertension due to small vessel vasculopathy. Cases of Budd–Chiari syndrome highlight that hepatic venous outflow obstruction can also occur, extending the spectrum of hepatic features.

No prior reports have documented focal nodular hyperplasia-like (FNH-like) lesions in DADA2, highlighting the novelty of this observation within the disease’s hepatic spectrum.

The underestimation of liver involvement probably arises from two factors: firstly, subtle or subclinical hepatic disease can be missed without systematic screening; secondly, the multisystem impact of DADA2 may overshadow hepatic symptoms in clinical practice. Nevertheless, the clinical significance is considerable. Portal hypertension carries risks such as variceal bleeding, hypersplenism, and reduced quality of life. Moreover, progressive liver disease may persist despite TNF inhibition, raising questions about the timing of treatment and the possible role of hematopoietic stem cell transplantation in refractory cases.

Comparative analysis with other systemic vasculopathies shows that hepatic involvement in DADA2 is unique in its frequent presentation as non-cirrhotic portal hypertension, rather than typical inflammatory hepatitis. This emphasises the vascular, rather than parenchymal, nature of hepatic injury in DADA2.

Nodular regenerative hyperplasia of the liver occurs in patients with the immunodeficiency phenotype of DADA2, as well as in those with hypogammaglobulinaemia disorders, particularly common variable immunodeficiency (CVID). NRH is the characteristic hepatic lesion in CVID, and relevant parallels with DADA2 have been drawn regarding this liver complication [8,14]. This relationship is also observed in our siblings. Only the one with significant hypogammaglobulinemia developed large regenerative nodules of focal nodular hyperplasia-like appearance (FNH-like), even though they share the same homozygous pathogenic mutations in the ADA2 gene.

Structural changes related to NRH can be detected on imaging studies such as ultrasound, CT or MRI; however, the radiologic findings are often nonspecific, and a definitive diagnosis typically requires clinical correlation and histopathologic confirmation [7,19,20]. FNH-like lesions, in contrast, exhibit more distinctive and characteristic imaging features than smaller regenerative nodules, which sometimes allow a confident radiologic diagnosis when interpreted in the proper clinical context [11,13].

Grey-scale ultrasound detection of NRH is challenging, particularly for an inexperienced sonographer, because nodules are usually small and commonly isoechoic, and they affect the entire hepatic parenchyma diffusely. Moreover, in our patient, many larger nodules were identified with CEUS, highlighting its additional value in evaluating such cases.

Contrast-enhanced ultrasound has been sporadically explored for evaluating NRH. Evidence is limited to isolated case reports [21]. Due to the diffuse and micronodular nature of NRH, the technique lacks sensitivity and specificity. CEUS may occasionally reveal non-specific or misleading enhancement patterns, so its findings should always be interpreted in conjunction with clinical data, cross-sectional imaging, and histopathological confirmation. Currently, no validated CEUS criteria are available for the identification of NRH.

Larger hepatic lesions, such as those resembling focal nodular hyperplasia (FNH), can be more accurately characterised using CEUS. These lesions typically exhibit intense centrifugal arterial-phase enhancement, followed by isoechoic enhancement during the portal venous and delayed phases [22]. The development of advanced CEUS techniques, together with higher-resolution contrast agents and emerging microbubble or molecular contrast media, is expected to improve the detection and characterisation of microvascular alterations seen in nodular regenerative hyperplasia (NRH).

In our patient, CEUS provided additional value in characterising the hepatic lesions. The presence of larger, hyperenhancing nodules with centrifugal arterial-phase filling and isoenhancement in the portal and late phases was consistent with FNH-like regenerative nodules. Depending on the clinical context, however, malignant lesions should be considered in the differential diagnosis.

Magnetic resonance imaging (MRI) supports the assessment of liver nodules. While more sensitive than ultrasound in detecting subtle parenchymal heterogeneity, MRI findings in NRH are often nonspecific, typically showing diffuse reticular or micronodular enhancement without a dominant mass or fibrous septa. These features help differentiate NRH from cirrhosis and focal nodular hyperplasia; however, histological confirmation remains crucial for an accurate diagnosis [7,20].

Conversely, larger or macro regenerative nodules (LRN) or FNH-like lesions show arterial hyperenhancement but no late-phase washout. They are typically similar in native and post-contrast features to FNH nodules, which are mostly found in adult women. Still, they are usually multiple, relatively homogeneous in size, and seem to have arterial flow overexpression as the leading cause. Usual MRI features of these nodules include: iso- to hypointensity on T2-weighted (T2w) images, iso- to hyperintensity natively on T1w images, no diffusion restriction, homogenous or “spoke-wheel” enhancement in the Gd-enhanced arterial phase, and no wash-out in the venous and late phase. A central scar with arterial hypoenhancement and late-phase enhancement can also be common [11,23]. The use of hepato-biliary contrast (i.e., Gadoxetic acid) will show either isoenhancement or hyperenhancement in the hepato-biliary phase, which can help distinguish from hepatocellular carcinoma or liver adenoma (where some wash-out is to be expected in the hepato-biliary phase) [11,13,24,25].

Considering current evidence, the most recent consensus recommends abdominal ultrasonography with Doppler as the primary imaging modality for evaluating the liver and spleen in DADA2. This approach reflects recognition that patients may develop noncirrhotic portal hypertension and vascular or inflammatory hepatic lesions, including focal nodular hyperplasia and nodular regenerative hyperplasia [26].

An MRI was also performed in our patient to provide a more detailed characterisation of the hepatic nodules. Multiple hyperenhancing focal lesions were identified during the arterial phase; most appeared isointense on T2-weighted and native T1-weighted images and iso- to hyperintense in the late phase, without evidence of washout. The nodules were relatively uniform in size and imaging appearance. Typical native and post-contrast features consistent with FNH were present in most lesions, except for two nodules that were T1 hypointense and T2 hyperintense. This finding suggests some heterogeneity among large regenerative nodules that do not always conform to classical imaging patterns. Given that these nodules also regressed during follow-up under therapy, they are unlikely to represent a different pathology. The native T1 hypointensity and T2 hyperintensity most likely reflect increased water content within the lesions, without a specific underlying cause.

Although suggested by magnetic resonance imaging or ultrasound scan, diagnosis must be histologically confirmed [14]. Being an invasive procedure with an increased risk of bleeding in the context of our patient’s thrombocytopenia and in the absence of severe liver damage, the liver biopsy was withheld in the first year after the diagnosis. After TNF inhibitors demonstrated effectiveness in reducing the number of nodules and their size, the procedure was no longer considered beneficial for the patient.

In the absence of histologic confirmation, alternative diagnoses were considered. Malignant hepatic nodules may complicate conditions associated with liver fibrosis (such as Budd–Chiari syndrome), but they are rare in autoimmune-related liver disease. Other benign entities, including hepatic hemangiomatosis or biliary hamartomas, were unlikely, as they would typically exhibit markedly hyperintense T2-weighted signals. The longitudinal evolution under Etanercept therapy, however, provides more substantial support for DADA2-related regenerative nodules.

Available data suggest that anti-TNF agents (Etanercept, Adalimumab, or Infliximab) significantly improve survival and overall disease control in DADA2, although their effect on hepatic involvement remains inconsistent. Data from the National Human Genome Research Institute shows that TNF inhibition decreases systemic inflammation and improves endothelial integrity in blood vessels, and may even reverse endothelial damage at the clinical, molecular, and histological levels [27].

While anti-TNF therapy has transformed the prognosis of DADA2 patients, its impact on hepatic manifestations remains uncertain. Some reports suggest stabilisation of liver disease with anti-TNF therapy, while others document progression of portal hypertension despite systemic disease control (Table 1) [4,6,28]. This raises the possibility that hepatic injury, once established, may be irreversible or only partially responsive to treatment.

One reported DADA2 patient exhibited clinical symptoms from early childhood, including primary vasculopathy, hypogammaglobulinaemia, a history of stroke, and liver disease [6]. He was diagnosed in his 40s and began Etanercept, which proved effective in controlling the disease activity. However, he died 18 months after his initial DADA2 diagnosis and one year after starting Etanercept, due to complications related to end-stage liver disease (nodular regenerative hyperplasia of the liver with portal hypertension and varices). In this case, the NHGRI group observed intact endothelial layers in postmortem brain and lung biopsies, suggesting that anti-TNF therapy was effective in those vascular territories. However, it does not demonstrate a definitive clinical effect on portal hypertension [27]. Overall, this case demonstrates the importance of early diagnosis and treatment to minimise organ damage and prevent life-threatening complications in patients with DADA2.

While a recent consensus on the evaluation and management of DADA2 patients emphasises the benefit of anti-TNF therapy for vascular/neurologic disease, the authors state that evidence for efficacy against non-vascular manifestations (including hepatic disease) is more limited [26].

In our patient, the FNH-like lesions showed an early positive response after starting Etanercept, with a noticeable decrease in both the size and number of nodules. This improvement was maintained, as imaging performed nine years into continuous anti-TNF therapy showed substantial regression of the hepatic nodules. To our knowledge, this is the first documented case of FNH-like liver lesions in DADA2 that show long-term improvement with anti-TNF treatment.

Available data—mostly from small cohorts and case reports—underscore the need for prospective, liver-focused monitoring of DADA2 patients receiving TNF inhibitors.

## 4. Conclusions

Hepatic vascular involvement represents a clinically significant yet frequently under-recognised component of the DADA2 phenotype. Diffuse nodular liver infiltration—consistently reported as regenerative nodular hyperplasia—has been described in some patients with the immunodeficiency phenotype of DADA2, underscoring the need for systematic liver evaluation. NRH-type involvement should be considered in individuals with abnormal liver function tests and features of portal hypertension, with biopsy required for confirmation.

Large regenerative nodules with an FNH-like appearance on CEUS or MRI have not previously been documented in this setting. In our patient, Etanercept therapy produced a favourable hepatic response, reflected by a significant reduction in both the number and size of the lesions. Our case contributes to the understanding of liver disease in DADA2 and the influence of imaging and treatment on the hepatic manifestations of the condition.

## Figures and Tables

**Table 1:** Anti-TNF therapy and hepatic outcomes in DADA2.

Study/Source	Year	No. of Patients	Hepatic Manifestation(s)	Effect of Anti-TNF Therapy	Comments/Notes
Barron et al. [4]	2021	60	Portal hypertension, nodular regenerative hyperplasia, increased liver stiffness	Mixed outcomes: some stabilisation, others progression despite systemic disease control	Liver stiffness improved in a few cases; portal hypertension persisted or worsened in others
Springer et al. [6]	2018	2 adult siblings	NRH, end-stage liver disease	One patient improved systemically; another developed irreversible hepatic failure	Suggests established liver damage may be refractory to TNF inhibition
Ombrello et al. [28]	2015	24 (NIH cohort)	Hepatomegaly, vascular involvement	Reported stabilisation in some hepatic findings; no detailed long-term data	Early report supporting TNF blockade as mainstay, without liver-specific endpoints
Lee et al. [26]	2023	Consensus/review	Multisystem vasculopathy (hepatic manifestations included)	Effective for vasculitic and hematologic features; hepatic response uncertain	Highlights lack of controlled data on hepatic disease progression
Deuitch et al. [27]	2022	Mechanistic study	endothelial cell model	TNF inhibition restores endothelial function in vitro	Supports biological plausibility, but no clinical hepatic outcomes reported

## Data Availability

The original contributions presented in this study are included in the article. Further inquiries can be directed to the corresponding author.

## Abbreviations

The following abbreviations are used in this manuscript:

DADA2	Deficiency of adenosine deaminase 2
FNH	Focal nodular hyperplasia
CEUS	Contrast-enhanced ultrasound
US	Ultrasound
MRI	Magnetic resonance imaging
TNF	Tumour Necrosis Factor
GGT	Gamma-glutamyl transpeptidase
NRH	Nodular regenerative hyperplasia
